# The anti-inflammatory and antioxidant effects of astaxanthin as an adjunctive therapy in community-acquired pneumonia: a randomized controlled trial

**DOI:** 10.3389/fphar.2025.1621308

**Published:** 2025-08-07

**Authors:** Fatma Makram Youssef, Hayam Ateyya, Amir Eskander Hanna Samy, Eman Mohamed Elmokadem

**Affiliations:** ^1^ Department of Pharmacy Practice and Clinical Pharmacy, Faculty of Pharmacy, Future University in Egypt, Cairo, Egypt; ^2^ Department of Critical Care, El Matarya Teaching Hospital, Cairo, Egypt

**Keywords:** community-acquired pneumonia, antioxidant, astaxanthin, oxidative stress, adjuvant therapy, inflammatory markers, SOFA, APACHE

## Abstract

**Background:**

Community-acquired pneumonia (CAP) is a leading cause of morbidity and mortality worldwide, particularly in low- and middle-income countries. Oxidative stress and excessive inflammation contribute significantly to disease progression and severity. Astaxanthin (ASX), a potent antioxidant and anti-inflammatory carotenoid, has demonstrated protective effects against oxidative damage and immune dysregulation in various conditions. However, its potential role as an adjunctive therapy in CAP remains underexplored. This study aims to evaluate the effects of ASX supplementation on inflammatory cytokines, and clinical outcomes in patients with CAP.

**Patients and methods:**

A prospective, randomized, double-blind, placebo-controlled study was conducted, in which adult patients diagnosed with CAP were enrolled and assigned to receive either 12 mg/day ASX or a placebo in addition to standard antibiotic therapy for 7 days. Inflammatory markers, including interleukin-6 (IL-6), tumor necrosis factor-alpha (TNF-α), and interleukin-10 (IL-10), were measured at baseline and post-treatment. Secondary outcomes included Sequential Organ Failure Assessment (SOFA) and Acute Physiology and Chronic Health Evaluation II (APACHE II) scores, as well as length of hospital stay.

**Results:**

A total of 80 patients (40 per group) completed the study. Patients receiving ASX exhibited significant reductions in pro-inflammatory cytokines compared to the placebo group. Notably, IL-6 and TNF-α levels were significantly lower in the ASX group at the end of the study (*P* < 0.05). Additionally, SOFA and APACHE II scores showed greater improvements in ASX-treated patients, suggesting a potential role in mitigating disease severity. Although the ASX group had a shorter hospital stay than the placebo group, the difference was not statistically significant (*P* > 0.05).

**Conclusion:**

ASX supplementation as an adjunct to standard CAP treatment significantly reduced inflammation while improving disease severity scores. ASX was found to be safe and well-tolerated. These findings highlight its potential therapeutic role in CAP management, warranting further investigation in larger, long-term clinical trials to confirm its benefits and establish optimal dosing strategies.

**Clinical Trial Registration:**

https://clinicaltrials.gov/study/NCT06334874, identifier NCT06334874.

## 1 Introduction

Community-acquired pneumonia (CAP) is the most prevalent form of pneumonia, occurring in individuals outside healthcare (Rider and Frazee, 2018). It remains a significant global cause of morbidity and mortality, particularly in low- and middle-income countries, where incidence and fatality rates are substantially higher than in high-income nations (Anderson and Feldman, 2023; Frigati et al., 2024; Atkinson and Mabey, 2025). In the United States, CAP is the leading infectious cause of death and one of the most common reasons for hospitalization, with an estimated 1.5 million hospitalizations annually and a readmission rate exceeding 9% (Kochanek et al., 2016; Ramirez et al., 2017; Dela Cruz et al., 2021).

CAP is caused by a diverse range of pathogens, including bacteria, viruses, and, less commonly, fungi. Among bacterial causes, *Streptococcus* pneumoniae is the most frequently identified pathogen, accounting for a significant proportion of cases across various severities. Other common bacterial culprits include *Haemophilus* influenzae, *Mycoplasma* pneumoniae, Chlamydophila pneumoniae, and *Legionella* species. Viral pathogens are also a substantial cause of CAP, with influenza A, rhinovirus, and respiratory syncytial virus being prominent. The specific pathogen responsible can influence the clinical presentation and severity of CAP, highlighting the importance of understanding the etiological landscape for effective management (Jain et al., 2015; Johansson et al., 2010; Musher et al., 2013).

The CAP pathogenesis involves a complex interaction between invading pathogens and the host immune system, often resulting in excessive inflammation and oxidative stress (OS) (Long et al., 2022). A dysregulated immune response, commonly referred to as a “cytokine storm,” plays a critical role in disease progression. Key inflammatory mediators such as interleukin-6 (IL-6) and tumor necrosis factor-alpha (TNF-α) drive excessive inflammation, while anti-inflammatory cytokines like interleukin-10 (IL-10) and interleukin-1 receptor antagonist (IL-1ra) attempt to counterbalance the response (Kumar, 2020; Keskinidou et al., 2022). However, in CAP, this equilibrium is frequently disrupted, leading to heightened inflammation and oxidative tissue damage (Quinton et al., 2018). The oxidative burden further amplifies inflammation and impairs immune function, perpetuating disease severity (Korkmaz and Traber, 2023; Barakat et al., 2023).

Conventional antibiotic therapy primarily targets the infectious component of CAP but does not directly modulate the inflammatory and oxidative pathways that contribute to disease progression (Muravlyova et al., 2016; Castillo et al., 2013; Gambini and Stromsnes, 2022). Consequently, there is growing interest in adjuvant therapies that mitigate inflammation, enhance immune response, and reduce OS (Gambini and Stromsnes, 2022; Mittal et al., 2014). Antioxidants have emerged as potential therapeutic agents due to their ability to neutralize reactive oxygen species (ROS), regulate cytokine production, and protect against lung tissue damage (Youssef et al., 2024; Lee et al., 2020).

Astaxanthin (ASX), a potent xanthophyll carotenoid derived from microalgae, is renowned for its powerful antioxidant, anti-inflammatory, and immunomodulatory properties (Mo et al., 2023). Its chemical structure, characterized by multiple hydroxyls groups, allows it to effectively neutralize ROS and reactive nitrogen species within cell membranes, providing robust protection against OS (Chang and Xiong, 2020; Pereira et al., 2021). Unlike other carotenoids, ASX’s unique ability to span biological membranes and scavenge free radicals positions it as an exceptional defense against cellular damage (Pereira et al., 2021).

Preclinical and clinical studies have highlighted ASX’s ability to modulate inflammatory pathways, enhance immune function, and protect against oxidative injury in various diseases (Kubo et al., 2019; Yiğit et al., 2019; Kim et al., 2011; Choi et al., 2011). ASX’s potent antioxidant action, coupled with its anti-inflammatory effects, makes ASX a promising therapeutic candidate for managing inflammatory diseases, such as CAP, where OS and inflammation are central to disease progression.

This study aims to evaluate the safety and efficacy of ASX as an adjuvant to standard antibiotic therapy in CAP. Specifically, it investigates the effects of ASX on inflammatory cytokines, and clinical outcomes to determine its potential role in reducing oxidative damage, modulating inflammation, and enhancing CAP management.

## 2 Patients and methods

### 2.1 Study design, setting, and ethical considerations

The current prospective, randomized, double-blind, placebo-controlled interventional study was conducted in accordance with the ethical principles of the Declaration of Helsinki. The study took place in the respiratory department of El Matarya Teaching Hospital, Cairo, Egypt, and included patients diagnosed with CAP. The study protocol was approved by the Research Ethics for Experimental and Clinical Studies, Faculty of Pharmacy, Future University in Egypt (REC-FPFUE-32/2023), as well as the General Organization for Teaching Hospitals and Institutes ethical committee (HM000167). Moreover, the study was registered on ClinicalTrials.gov (NCT06334874). The participants were oriented about the possible side effects of their treatment before taking part in the trial, and informed consent was acquired in compliance with the Declaration of Helsinki.

### 2.2 Methodology

#### 2.2.1 Study population

Eligibility screening was conducted for all participants recruited from the hospital’s respiratory department. Patients (≥18 years) diagnosed with CAP based on the American Thoracic Society/Infectious Diseases Society of America (ATS/IDSA) criteria were enrolled (Metlay et al., 2019). The diagnosis was confirmed by clinical symptoms (fever ≥38°C, cough, dyspnea, or pleuritic chest pain) and radiological findings.

Exclusion criteria included immunosuppression (HIV, immunosuppressive therapy), cancer, and recent ASX use (within 1 month). Patients with hypersensitivity to ASX, those on warfarin or antioxidant supplements, and those unable to comply with study procedures were also excluded. Additional exclusions encompassed pregnancy, lactation, age ≥70 years, severe comorbidities (e.g., tuberculosis, bronchiectasis, pulmonary edema), and severe pneumonia (CURB-65 ≥ 4).

#### 2.2.2 Study intervention and randomization

Eligible patients were randomly assigned to either the ASX or placebo group using a computer-generated randomization process. Group allocation was concealed in sealed envelopes to ensure blinding for patients, physicians, radiologists, and research personnel involved in evaluations, laboratory analysis, and serum collection.

##### 2.2.2.1 ASX Group

Patients received 12 mg once daily of oral ASX (NOW Foods, Egypt) alongside standard CAP treatment (Brendler and Williamson, 2019; Drugs.com, 2025; WebMD, 2025). The ASX used in this study was sourced from NOW Foods, Egypt, which exclusively utilizes natural ASX from AstaReal^®^. AstaReal^®^ is renowned for its stringent quality control measures, ensuring high purity, solvent-free, and standardized ASX derived from *Haematococcus pluvialis* microalgae cultivated under strictly controlled conditions (Foods, 2025).

##### 2.2.2.2 Placebo Group

Patients received a placebo identical in appearance to ASX, in addition to standard CAP treatment.

The study duration was 7 days. Both groups followed standard CAP therapy based on ATS/IDSA guidelines (Metlay et al., 2019).

#### 2.2.3 Study procedures

##### 2.2.3.1 Patient data collection

Baseline data, including demographic information (age, gender, height, weight, body mass index (BMI), complete medication history, and medical history, were recorded for all patients.

##### 2.2.3.2 Vital signs and physical examination

Daily medical assessments were conducted by physicians, while nurses monitored vital signs, including blood pressure (BP), body temperature (Temp), respiratory rate (RR), and heart rate (HR).

##### 2.2.3.3 Laboratory evaluations

Routine laboratory tests were performed daily, measuring hemoglobin (Hgb), blood glucose (BG), Liver enzymes: alanine transaminase (ALT) and aspartate transaminase (AST), kidney function markers: blood urea nitrogen (BUN) and serum creatinine (SCr), C-reactive protein (CRP), white blood cell (WBC) count, and red blood cell (RBC) count were assessed at baseline and after 7 days of therapy.

##### 2.2.3.4 Monitoring of adverse effects

Potential adverse effects of ASX supplementation, such as gastrointestinal discomfort, were tracked daily and documented using a standardized adverse event monitoring form.

##### 2.2.3.5 Severity assessment

Disease severity was evaluated using CURB-65, Acute Physiology and Chronic Health Evaluation II (APACHE II), and Sequential organ failure assessment (SOFA) scores to assess the extent of illness and predict clinical outcomes (Asai et al., 2019; Limapichat and Supavajana, 2022; Gursel and Demirtas, 2006). These scores were used to monitor disease progression and response to treatment over the study period.

##### 2.2.3.6 Radiological assessment

Chest radiography (CXR) scans were conducted before enrollment to confirm CAP diagnosis.

##### 2.2.3.7 Monitoring and follow-up

Patients were monitored daily for changes in CAP symptoms, treatment response, and overall clinical progression throughout the study period.

#### 2.2.4 Measurable outcomes

##### 2.2.4.1 Primary outcome

The primary outcome was the change in inflammatory markers, including TNF-α, IL-6, and IL-10 levels, between the ASX and placebo groups. These markers were measured at baseline and after 7 days of treatment to assess the effect of ASX supplementation.

Blood was collected, followed by centrifugation to separate Serum, which was subsequently stored at −80°C until analysis. Quantification of cytokine concentrations was performed using enzyme-linked immunosorbent assay (ELISA) kits in accordance with the manufacturer’s protocol (Elabscience company).

##### 2.2.4.2 Secondary outcomes

• Disease Severity Scores: APACHE II and SOFA scores were assessed at baseline and after 7 days of treatment in both groups.
• Length of Hospital Stay: The duration of hospitalization was compared between the ASX and placebo groups.

### 2.3 Sample size calculation, data management, and analysis

Sample size was determined using STATA, with a type I error (α) of 0.05 and a power (1-β) of 0.9. Based on prior research showing a significant reduction in IL-6 levels in the supplement group (8.91 ± 4.23 vs. 11.89 ± 1.62 in the placebo group) after 10 days of treatment, the required sample size was calculated as 26 patients per group (Abulmeaty et al., 2021). To account for potential dropouts, 40 patients were included in each group, totaling 80 participants.

Data were analyzed using IBM SPSS Statistics for Windows, Version 22.0 (IBM Corp., Armonk, NY). The Shapiro-Wilk and Kolmogorov-Smirnov tests were employed to assess normality. For parametric continuous variables, results are presented as means ± standard deviations, whereas non-parametric variables are shown as medians with interquartile ranges. Categorical data are expressed as frequencies and percentages. Between-group comparisons were performed using the unpaired Student’s t-test for normally distributed numerical data, and the Mann-Whitney U test for non-parametric data. Categorical variables were evaluated using either the chi-squared test or Fisher’s exact test. A *p*-value of <0.05 was deemed statistically significant.

## 3 Results

**FIGURE 1:**
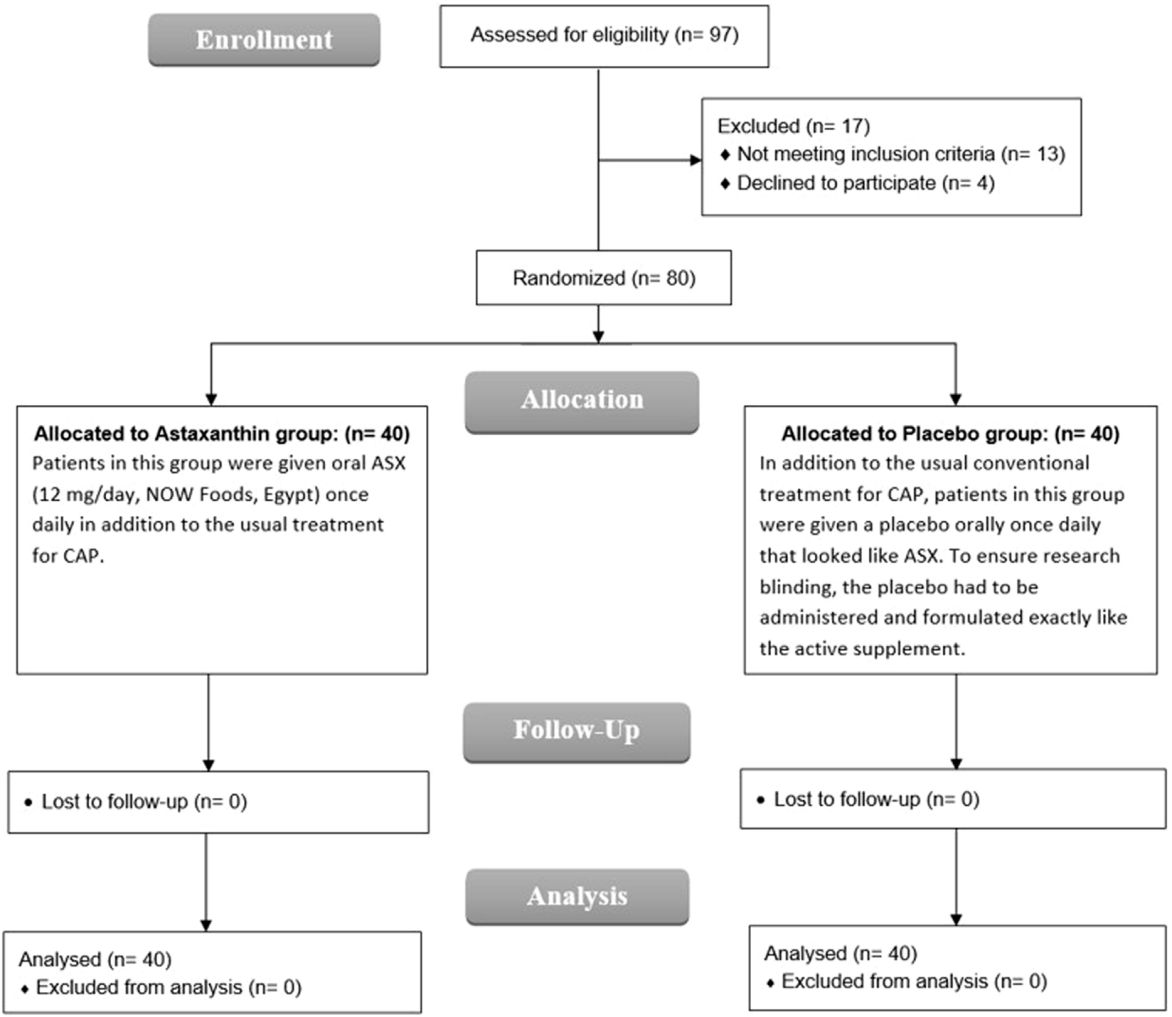
CONSORT flowchart.

Between April and October 2024, patient recruitment was carried out in the respiratory unit. A total of 97 individuals were screened for eligibility, of whom 80 met the inclusion criteria and were randomized in a 1:1 ratio to receive either ASX (n = 40) or placebo (n = 40). All enrolled participants completed the study without any dropouts and were included in the final analysis (Figure 1).

### 3.1 Baseline and demographic characteristics

As shown in Table 1, demographic data of the studied patients including age, height, weight, BMI, Temp and gender were insignificantly different between the two studied groups. The majority of patients (72.5% of ASX group vs. 77.5% of placebo group) showed no comorbidities, while 15% vs. 12.5% were diabetic and 12.5% vs. 10% were hypertensive, with no statistically significant difference between both groups. Moreover, the median CURB-65 score was 1 (IQR 0, 2) in the ASX group and 1 (IQR 1, 2) in the placebo group, and that difference was similar.

**TABLE 1:** Baseline characteristics of the studied groups.

Item	Astaxanthin group (n = 40)	Placebo group (n = 40)	P-value
Age (years)	51.6 ± 13.78	49 ± 10.66	0.348
Gender			
Male	19 (47.5%)	21 (52.5%)	0.655
Female	21 (52.5%)	19 (47.5%)	
Height (m)	1.66 ± 0.1	1.66 ± 0.09	0.953
Weight (kg)	65.4 ± 11.86	67.05 ± 10.04	0.504
BMI (kg/m^2^)	23.56 ± 2.38	24.23 ± 2.58	0.229
Temp on admission (°C)	38.81 ± 0.49	38.91 ± 0.43	0.374
Comorbidities			
No	29 (72.5%)	31 (77.5%)	0.872
DM	6 (15%)	5 (12.5%)	
HTN	5 (12.5%)	4 (10%)	
CURB-65 score	1 (0, 2)	1 (1, 2)	0.640

Continuous variables are expressed as mean ± standard deviation or median with interquartile range, as appropriate. Categorical variables are reported as frequencies and percentages. A *p*-value of less than 0.05 was considered statistically significant, BMI, body mass index; Temp, Temperature; DM, diabetes mellitus; HTN, hypertension; CURB-65, Confusion, Uremia, Respiratory rate, BP, Age ≥65 years.

### 3.2 Clinical parameters

As demonstrated in Table 2, there was no statistically significant difference between the two groups regarding HR, RR, and systolic BP. In terms of CXR, it evidenced bilateral lesions in most patients (65% of ASX group vs. 60% of placebo group), and no statistically significant difference was detected. The most frequently reported respiratory symptoms were sputum (72.5% of ASX group vs. 80% of placebo group), followed by dyspnea (70% vs. 75%) and pleuritic chest pain (55% vs. 62.5), and none showed a significant difference between both groups.

**TABLE 2:** Clinical parameters of the studied groups.

Item	Astaxanthin group (n = 40)	Placebo group (n = 40)	P-value
HR (bpm)	100.7 ± 2.5	99.68 ± 3.62	0.145
RR (breaths/min)	26.6 ± 1.72	26.03 ± 1.83	0.152
Systolic BP (mmHg)	128.13 ± 6.06	126.33 ± 11.46	0.384
Lesion on CXR			
Unilateral	14 (35%)	16 (40%)	0.644
Bilateral	26 (65%)	24 (60%)	
Respiratory symptoms			
Dry cough	2 (5%)	4 (10%)	0.675
Sputum	29 (72.5%)	32 (80%)	0.431
Dyspnea	28 (70%)	30 (75%)	0.617
Hemoptysis	2 (5%)	1 (2.5%)	>0.999
Pleuritic chest pain	22 (55%)	25 (62.5%)	0.496

Continuous variables are expressed as mean ± standard deviation or median with interquartile range, as appropriate. Categorical variables are reported as frequencies and percentages. A *p*-value of less than 0.05 was considered statistically significant, HR, heart rate; RR, respiratory rate; BP, blood pressure; CXR, chest radiography.

### 3.3 Hematologic parameters

**FIGURE 2:**
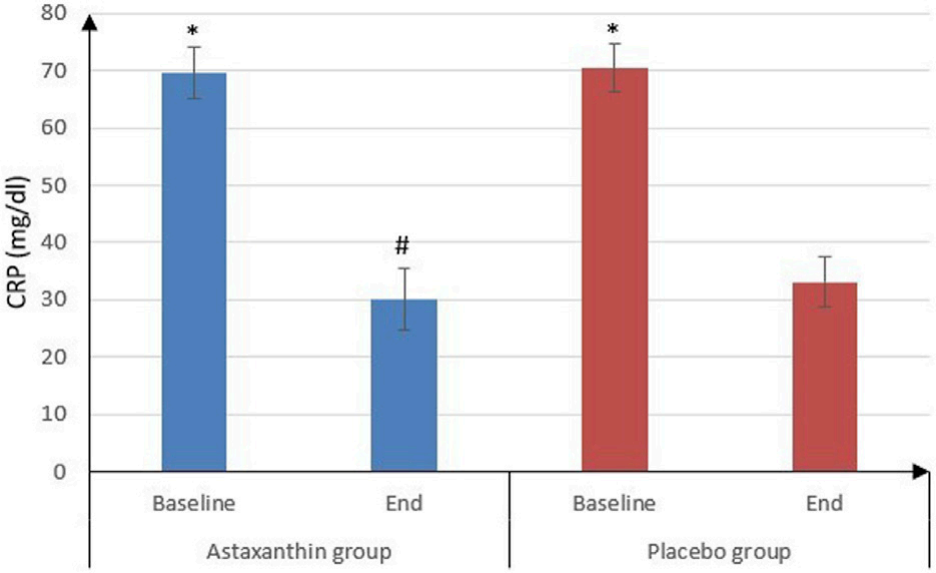
CRP of the studied groups.

No significant differences were observed between the ASX and placebo groups in most hematological parameters. BG levels decreased in the ASX group but with no statistically significant difference (*P* = 0.212) and it nearly remained unchanged in the placebo group (*P* = 0.685). Additionally, CRP levels significantly declined in both groups by the end of the study (*P* < 0.001), with a notably greater reduction in the ASX group compared to the placebo group (*P* = 0.007) (Table 3; Figure 2).

**TABLE 3:** Hematological parameters of the studied groups.

Item	Astaxanthin group (n = 40)	Placebo group (n = 40)	P-value
WBCs (10^3^/UL)			
Baseline	6.64 ± 0.93	6.9 ± 0.86	0.190
End	6.87 ± 1.07	7.01 ± 1.05	0.549
*P* value (Baseline vs. End)	0.205	0.542	
RBCs (10^3^/UL)			
Baseline	4.83 ± 0.66	4.73 ± 0.56	0.466
End	4.76 ± 0.56	4.77 ± 0.68	0.943
*P* value (Baseline vs. End)	0.297	0.596	
ALT (U/L)			
Baseline	36.5 ± 3.67	35.46 ± 4.89	0.138
End	35.21 ± 4.83	34.86 ± 4.12	0.258
*P* value (Baseline vs. End)	0.118	0.165	
AST (U/L)			
Baseline	38.2 ± 2.16	39.02 ± 2.19	0.097
End	36.52 ± 3.15	37.02 ± 1.93	0.394
*P* value (Baseline vs. End)	0.654	0.435	
BUN (mg/dL)			
Baseline	9.7 ± 0.7	9.5 ± 0.55	0.151
End	9.21 ± 0.68	9.23 ± 0.52	0.868
*P* value (Baseline vs. End)	0.143	0.015	
SCr (mg/dL)			
Baseline	0.45 ± 0.05	0.43 ± 0.04	0.063
End	0.42 ± 0.04	0.43 ± 0.03	0.366
*P* value (Baseline vs. End)	0.128	0.593	
Hgb (g/dL)			
Baseline	11.71 ± 1.01	11.65 ± 1.2	0.809
End	11.75 ± 0.84	11.67 ± 1.14	0.722
*P* value (Baseline vs. End)	0.672	0.825	
BG (mg/dL)			
Baseline	93 ± 10.09	92.03 ± 7.62	0.627
End	92.1 ± 8.56	91.7 ± 6	0.952
*P* value (Baseline vs. End)	0.212	0.685	
CRP (mg/dL)			
Baseline	69.51 ± 4.5	70.5 ± 4.17	0.312
End	30.05 ± 5.28	33.04 ± 4.33	**0.007**
*P* value (Baseline vs. End)	**<0.001**	**<0.001**	

Continuous variables are expressed as mean ± standard deviation or median with interquartile range, as appropriate. Categorical variables are reported as frequencies and percentages. A *p*-value of less than 0.05 was considered statistically significant, WBCs, White blood cells; RBCs, Red blood cells; ALT, alanine transaminase; AST, aspartate transaminase; BUN, blood urea nitrogen; SCr, Serum creatinine; Hgb, Hemoglobin; BG, blood glucose; CRP, C-reactive protein.

The bold numbers indicate statistically significant differences.

### 3.4 Inflammatory parameters

**FIGURE 3:**
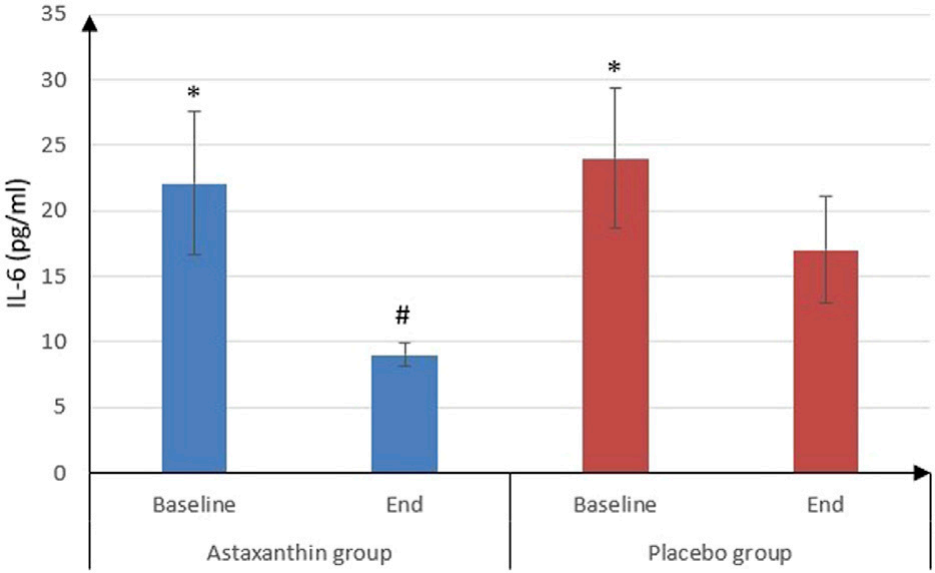
IL-6 of the studied groups.

**FIGURE 4:**
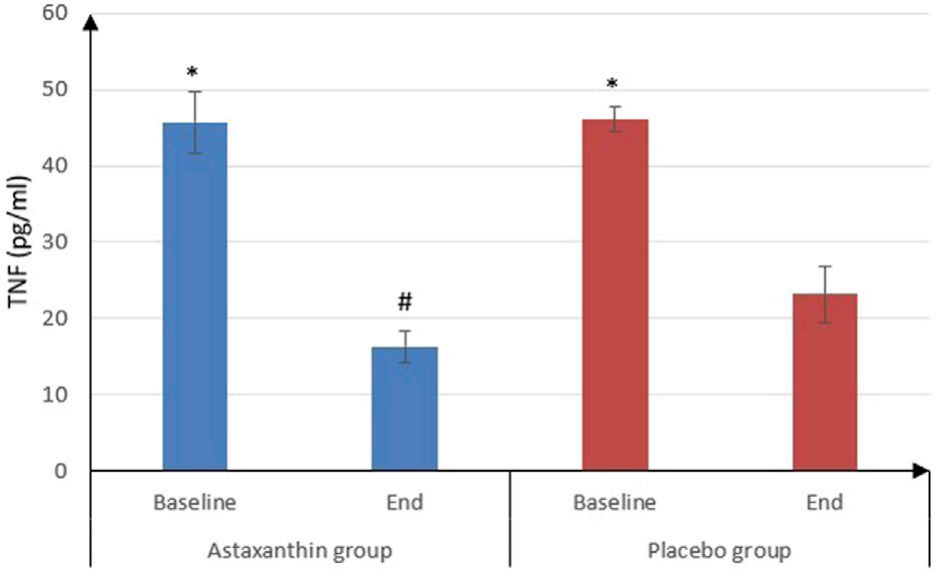
TNF -α of the studied groups.

IL-6 was significantly decreased from 22.09 ± 5.45 pg/mL at the start of study to 9 ± 0.9 pg/mL after treatment in ASX group (*P* < 0.001) and from 24.03 ± 5.38 to 17.02 ± 4.09 pg/mL in placebo group (*P* < 0.001), and that drop was deemed to be significantly more in ASX group (13.09 ± 5.41) than the placebo (7.01 ± 3.49), (*P* < 0.001). However, IL-10 values weren’t statistically significant between the start and end of the study in both groups. As for TNF-α, it was significantly decreased from 45.72 ± 4.03 to 16.28 ± 2.02 pg/mL in ASX group (*P* < 0.001) and from 46.11 ± 1.65 to 23.14 ± 3.64 pg/mL in placebo group (*P* < 0.001), and that decrease was significantly more in ASX group (29.45 ± 4.36) than the placebo (22.97 ± 3.99), (*P* < 0.001) (Table 4; Figures 3, 4).

**TABLE 4:** Inflammatory parameters of the studied groups.

Item	Astaxanthin group (n = 40)	Placebo group (n = 40)	*P*-value
IL-6 (pg/mL)			
Baseline	22.09 ± 5.45	24.03 ± 5.38	0.113
End	9 ± 0.9	17.02 ± 4.09	**<0.001**
*P* value (Baseline vs. End)	**<0.001**	**<0.001**	
Difference	13.09 ± 5.41	7.01 ± 3.49	**<0.001**
IL-10 (pg/mL)			
Baseline	1.11 ± 0.25	1.1 ± 0.28	0.847
End	1.14 ± 0.34	1.13 ± 0.29	0.910
*P* value (Baseline vs. End)	0.413	0.510	
Difference	−0.03 ± 0.22	−0.03 ± 0.3	0.953
TNF-α (pg/mL)			
Baseline	45.72 ± 4.03	46.11 ± 1.65	0.581
End	16.28 ± 2.02	23.14 ± 3.64	**<0.001**
*P* value (Baseline vs. End)	**<0.001**	**<0.001**	
Difference	29.45 ± 4.36	22.97 ± 3.99	**<0.001**

Continuous variables are expressed as mean ± standard deviation or median with interquartile range, as appropriate. Categorical variables are reported as frequencies and percentages. A *p*-value of less than 0.05 was considered statistically significant, IL, interleukin; TNF α -, tumor necrosis factor alpha.

The bold numbers indicate statistically significant differences.

### 3.5 Severity scores

**FIGURE 5:**
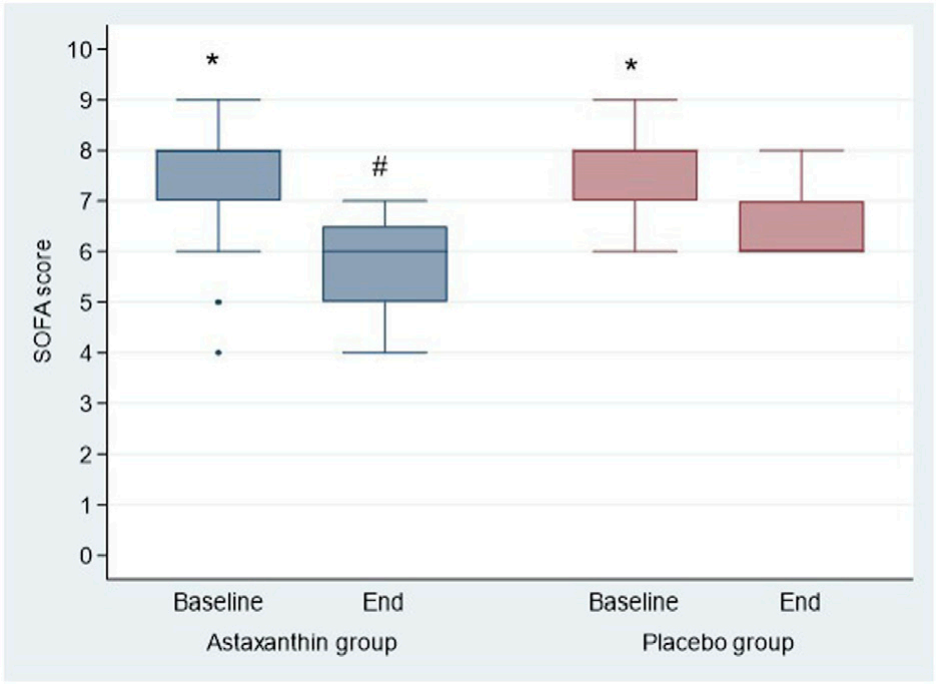
SOFA score of the studied groups.

**FIGURE 6:**
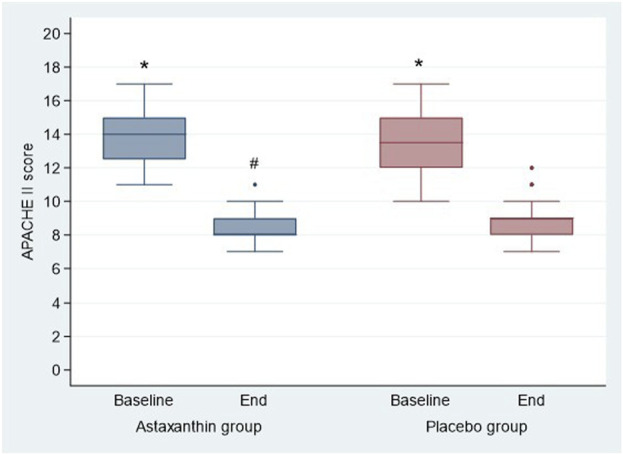
APACHE II score of the studied groups.

Regarding severity scores, the results showed a significant decrease in SOFA and APACHE II scores at the end of study than baseline in both groups (*P* < 0.001), and by comparing, both scores were significantly decreased in ASX group than the placebo only at the end of study (*P* = 0.005 and 0.032 respectively) (Table 5; Figures 5, 6).

**TABLE 5:** Severity scores of the studied groups.

Item	Astaxanthin group (n = 40)	Placebo group (n = 40)	*P*-value
SOFA score			
Baseline	8 (7,8)	8 (7,8)	0.279
End	6 (5, 6.75)	6 (6,7)	**0.005**
*P* value (Baseline vs. End)	**<0.001**	**<0.001**	
APACHE II score			
Baseline	14 (12.25, 15)	13.5 (12,15)	0.411
End	8 (8,9)	9 (8,9)	**0.032**
*P* value (Baseline vs. End)	**<0.001**	**<0.001**	

Continuous variables are expressed as mean ± standard deviation or median with interquartile range, as appropriate. Categorical variables are reported as frequencies and percentages. A *p*-value of less than 0.05 was considered statistically significant, SOFA, sequential organ failure assessment; APACHE, acute physiology and chronic health evaluation.

The bold numbers indicate statistically significant differences.

### 3.6 Hospital length of stay and medication safety

As shown in Table 6, the duration of hospital stay was shorter in the ASX group compared to the placebo group; however, this difference was not statistically significant. Moreover, no adverse effects were reported in ASX group throughout the study period.

**TABLE 6:** Hospital stay of the studied groups.

Item	Astaxanthin group (n = 40)	Placebo group (n = 40)	*P*-value
Hospital Length of stay (days)	7.2 ± 0.91	8.03 ± 1.44	0.541

Continuous variables are expressed as mean ± standard deviation or median with interquartile range, as appropriate. Categorical variables are reported as frequencies and percentages. A *p*-value of less than 0.05 was considered statistically significant.

## 4 Discussion

This randomized, double-blind, placebo-controlled trial is the first to evaluate the anti-inflammatory and antioxidant effects of ASX as an adjunctive therapy in hospitalized patients with CAP. The results demonstrate that ASX supplementation significantly reduced key inflammatory cytokines, improved clinical severity scores (SOFA and APACHE II), and was safe and well tolerated.

The findings are interpreted in relation to key clinical outcomes: inflammation, OS, disease severity, prognosis, and the potential mechanisms by which ASX may exert its effects.

### 4.1 Inflammation and cytokine modulation

Inflammation plays a central role in the pathogenesis and clinical progression of CAP. Elevated circulating levels of pro-inflammatory cytokines such as IL-6, TNF-α, and CRP are associated with increased disease severity and poorer outcomes (Siljan et al., 2018; Travlos et al., 2022). In the present study, ASX supplementation significantly reduced all three markers, indicating a substantial attenuation of systemic inflammation.

These findings are consistent with previous clinical and preclinical studies. Shokri-Mashhadi et al. reported that ASX supplementation significantly decreased IL-6 levels in patients with type 2 diabetes (Shokri-Mashhadi et al., 2021). Similarly, Cai et al. demonstrated that ASX inhibited lipopolysaccharide-induced IL-6 and TNF-α expression in lung tissue models (Cai et al., 2019). Rostami et al. observed reductions in inflammatory cytokines and OS in women with endometriosis following ASX use, and a meta-analysis of 14 clinical trials confirmed the efficacy of ASX in lowering CRP levels (Rostami et al., 2023; Xia et al., 2020).

Further supporting evidence comes from studies in other inflammatory contexts. For example, ASX administration has been shown to suppress age-related skin degradation and improve dermatologic outcomes by inhibiting inflammatory cytokine secretion from keratinocytes and reducing OS (Tominaga et al., 2017; Chalyk et al., 2017). Additionally, a randomized controlled trial by Fereidouni et al. found that ASX (6 mg/day for 8 weeks) significantly decreased IL-6 and IL-1β levels in infertile women with polycystic ovary syndrome (Fereidouni et al., 2024).

The anti-inflammatory action of ASX is thought to be mediated by inhibition of the Nuclear Factor kappa-light-chain-enhancer of activated B cells (NF-κB) and mitogen-activated protein kinase (MAPK) signaling pathways—key regulators of cytokine transcription (Wang and Qi, 2022). However, variability in outcomes has been observed across clinical contexts. For instance, studies involving patients with *Helicobacter pylori* infection and coronary artery disease reported no significant cytokine reductions, suggesting that ASX’s efficacy may depend on disease type, dosage, or treatment duration (Heidari et al., 2023; Andersen et al., 2007).

In our study, levels of IL-10, a key anti-inflammatory cytokine, remained unchanged following ASX supplementation. While preclinical studies have reported ASX-induced increases in IL-10, this effect has not been consistently observed in human trials (Balietti et al., 2016; Akhmad et al., 2025). These findings suggest that ASX primarily acts by suppressing pro-inflammatory mediators, rather than enhancing anti-inflammatory pathways (Wang and Qi, 2022). Such selective modulation may be especially advantageous in CAP, where excessive immune suppression could compromise pathogen clearance.

### 4.2 Oxidative stress and tissue protection

OS plays a critical role in the pathogenesis of CAP, contributing to alveolar damage and disease progression through the overproduction of ROS (Castillo et al., 2013). Although our study did not directly assess OS biomarkers, the significant reductions observed in IL-6, TNF-α, and CRP may reflect a downstream attenuation of oxidative-inflammatory cascades (Mittal et al., 2014; Biswas, 2016).

The antioxidant potential of ASX has been well documented in both clinical and preclinical studies. Choi et al. and Iwabayashi et al. reported improvements in malondialdehyde levels and total antioxidant capacity following ASX supplementation (Choi et al., 2011; Iwabayashi et al., 2009). In particular, Iwabayashi et al. observed enhanced redox balance in individuals under OS with a daily dose of 12 mg ASX, while Kim et al. demonstrated reduced lipid peroxidation in smokers (Kim et al., 2011; Iwabayashi et al., 2009). These findings are supported by meta-analyses from Hasani et al. and Ma et al., which concluded that ASX significantly increases total antioxidant capacity and enhances endogenous antioxidant enzyme levels, including superoxide dismutase, a key ROS-scavenging enzyme (Hasani et al., 2024; Ma et al., 2022).

Preclinical studies have further reinforced ASX’s antioxidant and cytoprotective capabilities. Research by Song et al. (2014), Zhou et al. (2015), Farruggia et al. (2018), Peng et al. (2020), Gao et al. (2019), and Xu et al. (2019) demonstrated that ASX reduces oxidative damage, modulates inflammatory pathways, and protects against cellular injury across a range of disease models (Song et al., 2014; Zhou et al., 2015; Farruggia et al., 2018; Gao et al., 2019; Xu et al., 2019).

ASX’s efficacy is largely attributed to its unique molecular structure, which enables it to span lipid bilayers and scavenge ROS both within and outside of cellular membranes (Ambati et al., 2014). This structural property is particularly relevant in CAP, where oxidative damage to the respiratory epithelium contributes to inflammation and pulmonary tissue injury (Xu et al., 2020).

In addition to direct ROS scavenging, ASX exerts antioxidant effects through regulation of cellular defense systems. One key mechanism involves the activation of nuclear factor erythroid 2–related factor 2 (Nrf2), a transcription factor that regulates antioxidant response elements and maintains redox homeostasis. Nrf2 is increasingly recognized as a therapeutic target in OS- and inflammation-related diseases. Furthermore, ASX has been shown to modulate mitochondrial function under OS conditions. Pre-treatment with ASX has been reported to restore mitochondrial membrane potential, reduce hydrogen peroxide-induced apoptosis, and enhance mitochondrial activity in redox-challenged states (Wang and Qi, 2022).

Taken together, these properties suggest that ASX may help preserve pulmonary epithelial integrity in CAP by attenuating oxidative injury and limiting secondary inflammatory damage. The clinical improvements observed in our study may, in part, be attributed to these antioxidant mechanisms.

### 4.3 Clinical severity scores (SOFA and APACHE II)

In this study, ASX supplementation resulted in a significant reduction in both SOFA and APACHE II scores compared to placebo. These scoring systems are well-established tools for assessing multi-organ dysfunction and predicting outcomes in patients with pneumonia and other critical illnesses (Asai et al., 2019; Gursel and Demirtas, 2006).

Our findings are consistent with previous research on antioxidant-based therapies in acute inflammatory conditions. For example, clinical trial evaluating several antioxidants in patients with COVID-19 and sepsis have shown that improvements in OS and inflammatory markers are associated with favorable changes in clinical severity scores (Chavarría et al., 2021). Similarly, studies in patients with OS–related conditions such as septic shock or those undergoing major surgery suggest that early antioxidant supplementation may reduce the risk of organ dysfunction and clinical deterioration (Aisa-Álvarez et al., 2023; Nathens et al., 2002).

The observed improvements in SOFA and APACHE II scores in our study may be attributed to the dual anti-inflammatory and antioxidant actions of ASX. Preclinical studies by Cai et al. support this hypothesis, demonstrating that ASX administration attenuated systemic inflammation and lung injury in experimental models of sepsis and endotoxemia conditions that share key pathophysiological features with severe CAP (Cai et al., 2019).

Collectively, these findings suggest that ASX may help preserve organ function, limit the progression of inflammatory injury, and reduce overall disease severity in hospitalized patients with CAP.

### 4.4 Prognosis and length of hospital stay

Although the reduction in hospital stay among patients receiving ASX was not statistically significant, a trend toward shorter duration was observed. This finding is consistent with previous studies evaluating antioxidant therapies in acute conditions such as acute pancreatitis and postoperative recovery, where early supplementation has been associated with reduced ICU stays and accelerated clinical improvement (Nathens et al., 2002; Sateesh et al., 2009).

Nonetheless, the evidence regarding the effect of antioxidant supplementation on hospitalization duration remains mixed. While some trials have reported meaningful reductions in hospital stay and enhanced recovery, others have found no significant differences (Alvi et al., 2023; Manzanares et al., 2012). These discrepancies may reflect differences in study design, patient characteristics, antioxidant formulations, dosages, and timing of administration.

In the context of our study, the absence of a statistically significant difference may be explained by the short duration of ASX administration (7 days) and the modest sample size, which may have limited the power to detect a true effect. Additionally, the timing of intervention relative to disease progression may have influenced the magnitude of clinical impact observed.

### 4.5 Comparison with other carotenoids

Several carotenoids, including β-carotene, lycopene, and lutein, have demonstrated antioxidant and anti-inflammatory effects in respiratory conditions such as asthma, chronic obstructive pulmonary disease, and cystic fibrosis. These compounds act primarily by scavenging ROS and modulating pro-inflammatory pathways (Kırkıl et al., 2012; Neuman et al., 2000; Çob et al., 2002; Sagel et al., 2018).

Among this class, ASX is widely regarded as the most potent, owing to its unique molecular structure. Its polar-nonpolar-polar configuration enables it to span lipid bilayers and neutralize ROS both within and outside cellular membranes—an advantage not shared by other carotenoids that tend to localize at the membrane surface (Ambati et al., 2014; Pfander, 1992).

Comparative studies have shown that ASX is up to 550 times more effective than vitamin E and substantially more powerful than vitamin C, lycopene, and green tea catechins in quenching singlet oxygen and other free radicals (Shimidzu et al., 1996; Ablon, 2021). This exceptional antioxidant capacity highlights ASX’s therapeutic potential in diseases characterized by intense oxidative and inflammatory stress, such as CAP.

Given the high oxidative burden and immune dysregulation associated with CAP, ASX’s superior efficacy relative to other carotenoids underscores its promise as a valuable adjunctive therapy in this setting.

### 4.6 Safety profile

ASX was well tolerated throughout the study period, with no adverse effects reported among hospitalized patients with CAP, including those in the acute phase of infection. This favorable safety outcome supports the potential use of ASX as an adjunctive therapy in acute inflammatory conditions.

These findings are consistent with previous clinical studies, including those by Capelli et al., Miyawaki et al., and Saito et al., which reported no significant adverse effects associated with ASX supplementation across a range of doses and treatment durations (Capelli et al., 2019; Miyawaki et al., 2008; Saito et al., 2012). Importantly, the absence of treatment-related side effects in our trial further confirms the excellent safety profile of ASX, even in acutely ill populations.

Compared to many conventional anti-inflammatory agents, which are often associated with gastrointestinal, renal, or cardiovascular side effects, ASX’s natural origin and strong safety record make it an especially attractive candidate for integration into supportive treatment regimens targeting inflammation and OS (Harirforoosh et al., 2013; Kahraman et al., 2023; Aly et al., 2024).

### 4.7 Limitations and future directions

Despite the positive findings of this study, some limitations should nevertheless be considered. The relatively small sample size may have limited the statistical power to detect differences in some outcomes and affects the generalizability of the findings. Larger, multicenter trials are needed to validate these results and confirm their clinical relevance.

Microbiological data on CAP pathogens were not collected. As different microorganisms can trigger distinct inflammatory responses, this limits our ability to assess pathogen-specific variations in ASX efficacy. Future studies should incorporate microbiological diagnostics to explore these differences.

A notable limitation of this study is the short follow-up duration, with outcomes assessed only over a 7-day period. While this timeframe offers insight into the acute effects of ASX on inflammatory and OS markers, it does not clarify whether these benefits are sustained beyond the immediate treatment window. Longer follow-up periods are needed to evaluate the long-term efficacy and safety of ASX as an adjunctive therapy in patients with CAP.

The fixed dose of 12 mg once daily may not have maintained optimal therapeutic levels throughout the day. Dose-ranging studies and evaluations of alternative regimens are needed to determine the most effective strategy.

Finally, gene expression analysis was not performed. While protein-level data were informative, molecular studies—such as RT-qPCR for cytokine mRNA—would offer deeper mechanistic insights. Future trials should integrate such analyses to better understand the transcriptional pathways affected by ASX.

## 5 Conclusion

Adjunctive ASX supplementation significantly attenuated systemic inflammation, as evidenced by reductions in IL-6, TNF-α, and CRP levels, and was associated with improvements in clinical severity scores in hospitalized patients with CAP. ASX was well tolerated, with no adverse effects reported. These findings support its potential role as a safe and effective adjunct to standard CAP therapy, meriting further investigation in larger, longer-term clinical trials to establish optimal dosing and assess sustained clinical benefits.

## Data Availability

The raw data supporting the conclusions of this article will be made available by the authors, without undue reservation.

## Glossary

CAP: Community-acquired pneumonia
OS: Oxidative stress
IL-6: Interleukin-6
TNF-α: Tumor necrosis factor-alpha
IL-10: Interleukin-10
IL-1ra: Interleukin-1 receptor antagonist
ROS: Reactive oxygen species
ASX: Astaxanthin
(ATS/IDSA): American Thoracic Society/Infectious Diseases Society of America
CURB-65: Confusion, Uremia, Respiratory rate, BP, Age ≥65 years
BMI: Body mass index
BP: Blood pressure
TEMP: Body temperature
RR: Respiratory rate
HR: Heart rate
Hgb: Hemoglobin
BG: Blood glucose
ALT: Alanine transaminase
AST: Aspartate transaminase
BUN: Blood urea nitrogen
SCr: Serum creatinine
CRP: C-reactive protein
WBC: white blood cell
RBC: Red blood cell
APACHE II: Acute Physiology and Chronic Health Evaluation II
SOFA score: Sequential organ failure assessment score
CXR: Chest radiography
ELISA: Enzyme-linked immunosorbent assay
DM: Diabetes mellitus
HTN: Hypertension
NF-κB: Nuclear Factor kappa-light-chain-enhancer of activated B cells
MAPK: Mitogen-activated protein kinase
Nrf2: Nuclear factor erythroid 2–related factor 2.
